# Prescriptions of Traditional Chinese Medicine Are Specific to Cancer Types and Adjustable to Temperature Changes

**DOI:** 10.1371/journal.pone.0031648

**Published:** 2012-02-16

**Authors:** Pei-Hsun Chiu, Hsin-Ying Hsieh, Sun-Chong Wang

**Affiliations:** 1 Institute of Systems Biology and Bioinformatics, National Central University, Chungli Taoyuan, Taiwan; 2 Epigenetics Laboratory, Centre for Addiction and Mental Health, Toronto, Ontario, Canada; The University of Kansas Medical center, United States of America

## Abstract

Targeted cancer therapies, with specific molecular targets, ameliorate the side effect issue of radiation and chemotherapy and also point to the development of personalized medicine. Combination of drugs targeting multiple pathways of carcinogenesis is potentially more fruitful. Traditional Chinese medicine (TCM) has been tailoring herbal mixtures for individualized healthcare for two thousand years. A systematic study of the patterns of TCM formulas and herbs prescribed to cancers is valuable. We analysed a total of 187,230 TCM prescriptions to 30 types of cancer in Taiwan in 2007, a year's worth of collection from the National Health Insurance reimbursement database (Taiwan). We found that a TCM cancer prescription consists on average of two formulas and four herbs. We show that the percentage weights of TCM formulas and herbs in a TCM prescription follow Zipf's law with an exponent around 0.6. TCM prescriptions to benign neoplasms have a larger Zipf's exponent than those to malignant cancers. Furthermore, we show that TCM prescriptions, via weighted combination of formulas and herbs, are specific to not only the malignancy of neoplasms but also the sites of origins of malignant cancers. From the effects of formulas and natures of herbs that were heavily prescribed to cancers, that cancers are a ‘warm and stagnant’ syndrome in TCM can be proposed, suggesting anti-inflammatory regimens for better prevention and treatment of cancers. We show that TCM incorporated relevant formulas to the prescriptions to cancer patients with a secondary morbidity. We compared TCM prescriptions made in different seasons and identified temperatures as the environmental factor that correlates with changes in TCM prescriptions in Taiwan. Lung cancer patients were among the patients whose prescriptions were adjusted when temperatures drop. The findings of our study provide insight to TCM cancer treatment, helping dialogue between modern western medicine and TCM for better cancer care.

## Introduction

According to the World Health Organization (WHO), cancer is the number one cause of mortality worldwide, accounting for 7.6 million deaths, or 13% of all deaths, across the globe in 2008 [1]. The toll is expected to rise continuously to over 11 million in 2030. Environmental factors are believed to be a primary contributor to the pathogenesis. The hazards range from physical agents such as ionizing and ultraviolet radiations, chemical agents such as dioxins and arsenic, to biological agents such as human papillomavirus and hepatitis B virus. Other risk factors include smoking, alcoholism/diet, obesity, ageing and genetics. Cancer cells, with mutated genomes, share three characteristics: uncontrolled cell multiplication, invasion of adjacent tissues, and migration to non-adjacent sites [2]. Metastasis of cancer, usually via bloodstream or lymphatics, to other vital organs such as lungs, liver, brain and bones adds to the malignancy and worsens the prognosis of the disease.

Treatment of cancer in modern western medicine includes surgery, radiotherapy and chemotherapy. Treatment modality depends on the site of cancer origin and stage of cancer progression, and typically involves a combination of modalities, for example a surgical removal followed by radiation or chemotherapy. Radiotherapy kills cells by breaking the DNA with X-, gamma-rays or charged particles and the free radicals generated in the radiation. Cytotoxic chemotherapy tames cells by stopping their division with small molecules that stop cell cycle or DNA synthesis. The two therapies seldom eliminate all cancer cells as, at increasing dosages, more nearby healthy and normal fast growing cells are compromised. A new and encouraging development in the last 15 years is targeted therapy which employs small molecules or monoclonal antibodies that bind and block the functions of the overly expressed genes in cancer cells [3], [4]. Examples of the inhibited targets are tyrosine kinases, vascular endothelial growth factors (VEGF) and histone deacetylases (HDAC) that are involved in growth signaling, angiogenesis and epigenetic regulation, respectively, of the cancer cells.

The outcome of the “war on cancer”, initiated 40 years ago by the United States administration, has been debated in public media. According to a latest analysis by the American Cancer Society, the overall age-adjusted death rate of all cancers in men (women) dropped by 11% (6%) to 2.2 (1.5) per 1000 in the United States in 2006, compared to that in 1970 [5]. The declines were however largely attributed to better prevention and early detection including reduced smoking and increased mammogram and Pap tests, suggesting room for further development of new, complementary and alternative (CAM), as well as integrative cancer therapies. Targeted therapy may illustrate the trend of development. The effectiveness of targeted therapy was found to vary from patient to patient, depending on the carried cancer subtypes [6], [7]. Furthermore, combining a drug targeting cell proliferation and a drug targeting angiogenesis may enhance the effect of the treatment and ameliorates the issue of drug resistance [8]. Neither the concept of personalized medicine nor the prescription of drug mixtures is new to traditional Chinese medicine (TCM) [9], [10], a CAM that originated in China two thousand years ago and still thrives in far east Asia today.

What cancers are in TCM is best revealed from TCM prescriptions to cancers. Toward a systematic and scientific investigation of TCM treatment of cancers, the elements in TCM prescriptions have to be established and standardized, similar to the consideration of genes as the fundamental elements in genomics. In a typical TCM prescription to a patient can be found, for example, two TCM formulas and four TCM herbs. TCM formulas are believed to evolve from synergistic combinations of multiple TCM herbs. Many TCM formulas from authoritative TCM classics [11], with specified ingredient herbs and relative weights, stand the test of time and are still highly received today. The chemical composition within an herb can change depending on the harvest times/regions and processing methods. Minimizing the variability in the chemical profiles of the herbs/formulas can be relegated to certified manufacturers of TCM medicinals. A certification system involving government, for example, mandatory GMP compliance, also helps eliminate the concern of herbal toxicity due to heavy metal, pesticide and microbiological contaminations [12]. A TCM prescription *p* is then represented by *p* = *a_1_ m_1_*+*a_2_ m_2_*+…+*a_N_ m_N_*, where *m_i_* can be either a TCM herb or a classical TCM formula, *a_i_* a numeric value between 0 and 1 for the percentage weight of *m_i_* in the prescription, and *N* the number of *m_i_* in the prescription so that *a_1_*+*a_2_*+…+*a_N_* = 1. The representation is simple yet practical as prescriptions by a TCM doctor to patients of the same western, molecularly diagnosed, disease can contain exactly the same herbs/formulas *m_i_*'s and *N* but different weights *a_i_*'s, one of the manifestations of personalized medicine in TCM

TCM has developed its own system of diagnostics or TCM syndrome differentiation via for example tongue and pulse readings. Outcomes of TCM diagnoses in TCM terms could lack of consistency among TCM doctors [13], [14]. As modern western medicine has become an integral part of the core curriculum of the TCM education in Taiwan, diagnosis made in western terms, i.e., the International Classification of Diseases codes (ICD-9), has been enforced for the reimbursement of TCM prescriptions to the public health insurance program in Taiwan. With a large quantity of diagnosis and prescription data, we are then able to statistically associate an ICD-9 coded cancer to a TCM prescription: *p*(cancer ICD9 code) = *a_1_ m_1_*+*a_2_ m_2_*+…+*a_N_ m_N_*. ICD-9 codes on one side of the association and *a_i_*'s on the other side can also be regarded as a bridge between modern western medicine and traditional Chinese medicine.

A mapping from TCM prescriptions to cancers sheds light on cancer therapy as TCM, as well as many other ancient medicines, is believed to be holistic [15]. On the other hand, mapping cancers to TCM prescriptions allows a better understanding of TCM as cancer biology has been extensively and rigorously investigated at the molecular and cellular levels. Specifically, with a year's worth of collection of 187,230 TCM prescriptions to over 30 cancer types in Taiwan in 2007, the questions we would address include: what are the most common TCM formulas and herbs for all and individual cancers? How are the relative weights of the individual formulas and herbs in a prescription distributed? Are the TCM prescriptions different for different cancers? How cancer type-specific are they? What are the possible mechanisms of actions, at cellular level, of *harmonizing* TCM formulas in comparison to *tonifying* TCM formulas? What diet is potentially beneficial for cancer patients? Do TCM cancer prescriptions change with seasons? If so, what cancer patients are susceptible to which meteorological variables?

## Materials and Methods

### Ethics Statement

No informed consent was required because the data were analyzed anonymously.

### National Health Insurance Research Database (Taiwan)

Every citizen of Taiwan is under the National Health Insurance program, which is a public, single-payer insurance plan covering many treatments including outpatient TCM treatment. When registered healthcare providers file a reimbursement to the Bureau of National Health Insurance (BNHI), the original claim including diagnosis and prescription is submitted. We applied for and obtained from BNHI [16] the complete TCM claims made throughout Taiwan in 2007. Note that information on personal identification in the data was scrambled before its release to researchers so that patient privacy is protected. From the number of distinct individuals ( = 6,609,872) in the TCM data and population of Taiwan in 2007, we estimated that 28.8% of the Taiwan population patronized TCM in 2007. Note that if we include self-pay TCM treatments which are not in the reimbursement data, the prevalence should rise. From the total number of claims, we estimated the average number of visits per patient per year to be 5.3. The diagnosis column in the data can have up to three ICD-9 codes, for the primary, secondary and tertiary diagnosis of the patient during that clinic visit. We focus on cancers by limiting the primary ICD-9 codes to be within 140 and 239, which are the codes allocated for neoplasms by the International Classification of Diseases published by the WHO. The resulting 187,230 cancer diagnoses and the corresponding TCM prescriptions are the object of current study.

### Data analysis

#### Cancer-Herb Data Matrix

A repertoire of classical TCM formulas and single TCM herbs in various forms (e.g. powder, pill) derived from concentrated herbal extracts manufactured by certified, GMP-compliant TCM suppliers were approved and used in Taiwan. Potency changes with forms according to TCM [17] and we found the numbers of formulas and herbs including their different forms to be 336 classical formulas and 410 single herbs as of 2007. A numeric matrix of dimensions 746 by 100 was created, where 746, the number of rows, comes from the number of reimbursable TCM formulas (i.e. 336) plus the number of reimbursable TCM herbs (i.e. 410), and the number of columns 100 is the number of cancers from the 3-digit ICD-9 codes from 140 to 239 designated for neoplasms. The cancer-herb matrix was initialized to zero. For every claim, the percentage weight *a_i_* of each TCM formula or herb in the prescription was calculated and added to the corresponding cell of the cancer-herb matrix. The procedure iterated over the 187,230 claims. We then divided the column by the frequency of the cancer to adjust for the effect of different cancer occurrences. Note that in the preparation for such matrices, we had two scenarios: i) the prescriptions for those which have only primary but have neither secondary nor tertiary diagnosis (No. of claims = 100,679); and ii) those which have primary cancer and any secondary but none tertiary diagnosis (No. of claims = 51,275). The purpose of the second scenario was to study prescription changes due to a secondary morbidity.

A TCM formula delivers one of the 21 categories of TCM therapeutic effects according to TCM [18]. We show in Table S1 the TCM categories and numbers of formulas in each of the categories. In the study of TCM category and cancer, a 21 by 100 cancer-category data matrix was created from the 187,230 claims in the same way described above for the cancer-herb data matrix. A cancer-nature matrix and a cancer-flavour matrix were also prepared the same way for the studies of TCM natures and TCM flavours and cancers. Information and assignment of TCM categories to formulas and TCM natures and flavours to herbs were as previously described [19]. The numbers of reimbursable herbs in each of the TCM natures and flavours are shown in Tables S2, S3.

#### Hierarchical Clustering

A technique in statistical data analysis and bioinformatics that identifies i) subsets of observations called clusters; and ii) the hierarchical relations among the clusters [20]. The result of the hierarchy of clusters is usually presented in a tree-like diagram. The observations in the current application are the cancer-herb data matrix prepared above. Specifically, when the weight distributions of the 746 TCM formulas and herbs to two cancers are similar, the two cancers are considered to be in a cluster. Similarly, if the weight distributions of two formulas (two herbs, or one formula and one herb) across the 100 cancers are similar, the two formulas (two herbs, one formula and one herb) are said to be clustered. The algorithm runs bottom-up to grow the cluster and build the hierarchy of the clusters along the way. We used the heatmap function in R [21] with the default parameter setting for the hierarchical clustering analysis.

#### Principal Component Analysis

A method of exploratory data analysis that transforms the original high dimensional data into a low dimensional space while preserving most of the information, i.e. variance, in the data [20]. In the present case, a cancer is characterized by 746 formulas and herbs. Two herbs may be correlated because their weight distributions across the 100 cancers are identical or nearly identical. We may then replace the two herbs by using the mean of the two without losing much information. The dimension of the data, i.e. 746, is then reduced by one. Principal component analysis (PCA) is a procedure that identifies and constructs the uncorrelated axes, called principal components (PCs), using linear combinations of the original and usually highly correlated axes, in achieving dimension reduction. Note that we order PCs so that cancers spread out in the space of the first few, usually 2 or 3, PCs. We used the prcomp function in R [21] with the default parameter setting for the PCA analysis.

## Results

We start with cancers which are not accompanied with any secondary morbidities, the number of such cases totalling 100,679 in 2007. The female and male proportions are 69% and 31% while the distributions of ages (at diagnosis) are 47±13 and 56±16 years old as shown in Figure S1. The age distributions for both sexes slightly tilt to the old age as age is known to be a risk factor for the disease. Cancers differ by their occurrences. As shown in the cancer frequency distribution of Figure S2, uterine leiomyoma ranks number one, followed by female breast cancer, liver cancer and nasopharyngeal cancer. The top four cancers account, respectively, for 21%, 15%, 6% and 4% of all the cancer diagnoses. The top two cancer types explain the predominance of female TCM patients in Taiwan. The high frequency of nasopharyngeal cancer mirrors the known epidemiology of high nasopharyngeal carcinoma occurrence in the regions of southern China and Taiwan [22]. In the rest of the study, we focus on the top 30 cancers of Figure S2, which account for 90% of all the cancer diagnoses in the data.

### Zipf-like distribution of the TCM formulas and herbs to cancers

**Figure 1:**
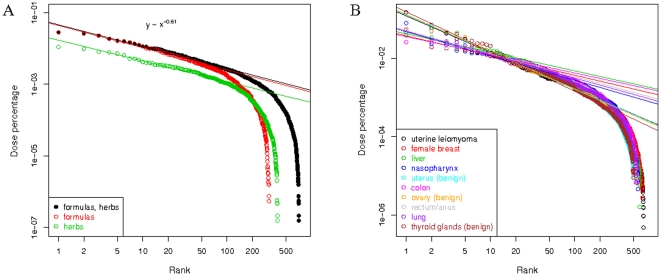
Zipf distributions of TCM formulas and herbs to cancers. The percentage weight of a formula or herb in a prescription is calculated. The weights are averaged over individual cancers, and then over the 30 cancer types. Formulas and herbs are ranked according to the averaged weights. (A) Weights averaged over the 30 cancers are plotted against their ranks. Note of the log scale of the axes. Lines are linear regressions to their top 50 formulas and/or herbs. The slopes of the lines give the Zipf exponents. (B) Weights over individual cancers are plotted against their ranks. Lines are linear regression fits to their top 20 formulas and herbs.

The average percentage weight *a_i_* of a formula or herb in a prescription, normalized for the cancer frequency, was obtained as described in Material and Methods. We rank the formulas and herbs according to the weights *a_i_*'s and plot weights against ranks in Figure 1A. For the top ranking formulas and herbs, we observe a power-law decrease of weight with rank: weight ∼rank^−*β*^, where *β* = 0.61. In many other disciplines of sciences, including natural, social, economical and biological sciences, similar behaviours were found with *β* close to 1 and the distribution is called Zipf's law [23]. Prominent examples include the frequencies of words and phrases in a book [24] and the sizes of firms in the United States [25]. The analysis was also done on individual cancers as shown in Figure 1B. We found that benign neoplasms have a statistically significantly larger Zipf exponent *β* = 0.80 than malignant cancers *β* = 0.54 (*t*-test *P*-value = 0.002 and Figure S3). For *β* = 0.80 (0.54), the relative weights of the top 5 formulas/herbs go like: 1.00 (1.00), 0.57 (0.69), 0.42 (0.55), 0.33 (0.47) and 0.28 (0.42). A smaller *β* may therefore indicate a less consensus, as a consequence of the complexity of the disease, among the TCM doctors on the treatment of malignant cancers.

### Specificity of TCM prescriptions to cancer sites

**Figure 2:**
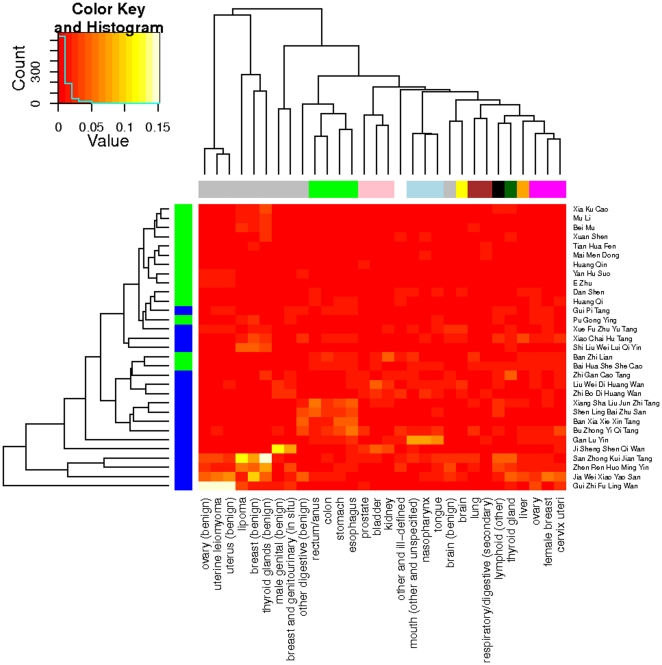
Heatmap and hierarchical clustering of cancers and TCM formulas/herbs. Cancers similar in terms of the prescribed TCM formulas and herbs are clustered and shown within the branches of the tree on the top. Horizontal color stripes encode cancer types. For example, benign neoplasms are in gray. Formulas and herbs that are similar in terms their usage across the cancers are clustered and shown on the left tree. Blue color in the vertical color bar indicates formulas while green indicates herbs. The heatmap shows only the 30 heaviest formulas/herbs. Color key and histogram show the value and distribution of the percentage weights. Figure S4 is the heatmap with all the 746 formulas and herbs.

Benign neoplasms do not metastasize, distinguishing themselves from malignant ones. A different Zipf exponent of the formulas/herbs is intriguing. To look for further differences, we performed a hierarchical clustering of the cancer-herb data matrix. The results, on the top dendrograms of Figures 2 and S4, show that benign neoplasms, based on similarity in the prescribed formulas and herbs, were grouped together into clusters by the algorithm, reconfirming the result of Zipf analysis. Moreover, malignant cancers which are proximal in their anatomical positions (e.g. mouth, tongue and nasopharynx) or similar in the physiological functions (e.g. esophagus, stomach, colon and rectum) cluster together. Since the clustering of cancers was based on the profiles of percentage weights, the result indicates that, through weighted combinations of TCM formulas and herbs, TCM cancer prescriptions are specific to anatomical sites and physiological functions.

On the left dendrograms of Figures 2 and S4 show the clustering of the formulas and herbs based on their weights across the different cancers. The result indicates that TCM formulas play a more significant role than single herbs in a prescription as they weigh relatively heavier than single herbs in the prescription. Further analysis shows that a TCM cancer prescription consists on average of 2.2±1.2 formulas and 4.0±2.8 herbs and that the average (median) weight of a formula is 2.4±0.4 (1.2±0.8) times the average (median) weight of an herb in the prescription. The practice of combining formulas and herbs echoes the reported pattern of TCM co-prescriptions to acute nasopharyngitis in Taiwan [26].

### TCM cancer prescriptions are *tonifying*, *harmonizing* and *fire-purging*

**Figure 3:**
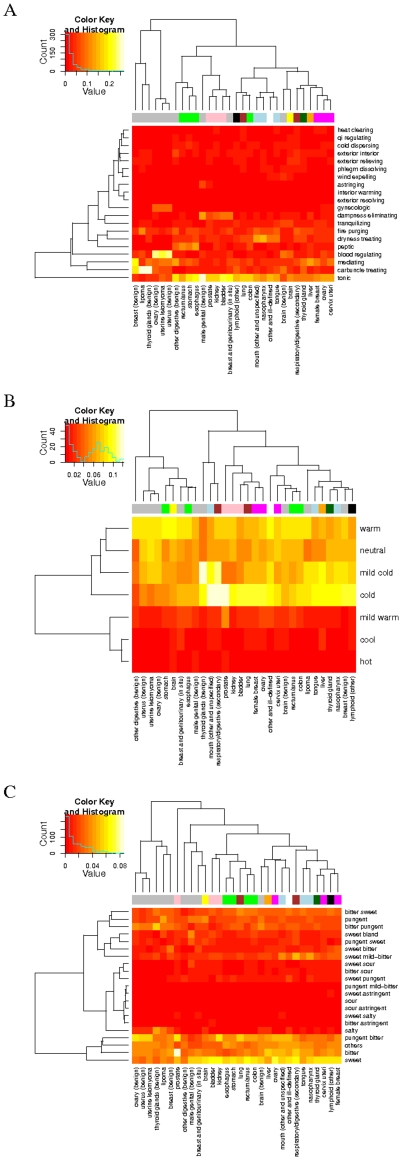
Heatmap and hierarchical clustering of cancers and TCM categories, natures, and flavors. The effect of a TCM formula is classified into one of the 21 TCM categories. The average percentage weight of a category in the prescriptions to each cancer is calculated. In (A) shows the heatmap of the cancer by category weights. Similarly, an herb is designated its TCM nature and TCM flavor. The heatmaps of the cancer by nature and cancer by flavor weight matrices are shown in (B) and (C).

It is interesting to learn more about cancer care from TCM perspectives. A TCM formula is traditionally classified into one of 21 TCM therapeutic categories [18]. Given that TCM formulas are important players in TCM cancer prescriptions, we ranked the TCM categories of the TCM cancer formulas. The top five most common categories for malignant cancers were found to be *tonifying*, *mediating* (or *harmonizing*), *dryness-treating*, *fire-purging*, and *peptic*. Furthermore, the clustering result in Figure 3A reveals TCM treatments combining *tonifying*, *mediating* and *fire-purging* formulas to most of the malignant cancers while *dryness-treating* formulas to nasopharyngeal and mouth cancers and *peptic* formulas to esophagus, rectum and stomach cancers. For benign neoplasms, the top five categories were *carbuncle-treating*, *blood-regulating*, *tonifying*, *mediating* and *fire-purging*. *Carbuncle-treating* formulas were common to lipoma and *blood-regulating* formulas to uterus and ovary neoplasms, as shown in the figure.

Single herbs in a TCM prescription play supporting roles according to TCM principles, which our weight analysis above also corroborates. Herbs spread out under the prism of TCM *cold-hot* nature [19], [27]. We ranked and clustered the TCM natures of the herbs in TCM cancer prescriptions as shown in Figure 3B. For malignant cancers, the top four natures were found to be *cold*, *warm*, *mild-cold* and *neutral*, suggesting that *cold* herbs enhance the *fire-purging* formulas in the prescriptions. On the other hand, the top four TCM natures for benign neoplasms were found to be *warm*, *mild-cold*, *cold* and *neutral*, suggesting *warm* herbs to enhance the *blood-regulating* formulas. In addition to TCM natures, singles herbs carry their TCM flavors based on the principles of TCM [19], [27]. A similar analysis in Figure 3C shows *sweet*, *pungent-bitter* and *bitter* as the top three flavors for both malignant and benign neoplasms.

### Adjustment of TCM prescriptions to secondary diagnoses

**Figure 4:**
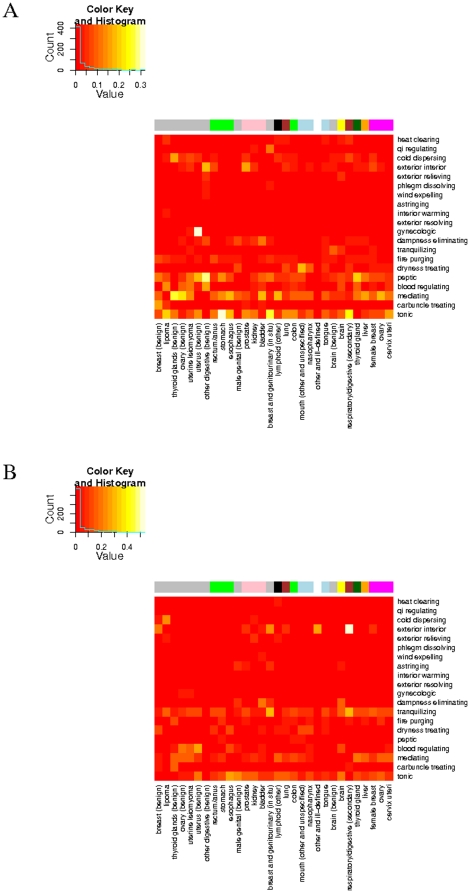
Heatmap of TCM categories and cancers with a secondary morbidity. Orderings of cancers and categories follow those of Figure 3A for easy comparison. (A) The secondary diagnosis is stomach disorder, ICD-9 = 5369. (B) The secondary diagnosis is sleep disturbance, ICD-9 = 7805.

The analysis has so far been on prescriptions to cancer patients without other complications. We identified 51,275 diagnoses which have a secondary ICD-9 in addition to their primary cancer ICD-9. The sex and age distributions of these patients are 62% (female), 38% (male) and 51±13 (female), 58±15 (male) years old as shown in Figure S5. The most common secondary morbidities are found to be stomach functional disorder, ICD-9 = 5369, and sleep disturbance, ICD-9 = 7805, as shown in the secondary ICD-9 distribution in Figure S6. We performed clustering analysis on the data derived from the 51,275 claims to find that *peptic* formulas become the third common formulas after *tonifying* and *mediating* for the treatment of all the primary cancer diagnoses with stomach functional disorder as the accompanying secondary diagnosis as shown in Figure 4A. Similarly, the top three TCM categories for the cancers with sleep disturbance as the secondary diagnosis are found, in Figure 4B, to be *tonifying*, *tranquilizing* (or *sedative*) and *mediating* formulas. We conclude that TCM prescriptions to cancers with secondary mobility are adjusted by the incorporation of relevant TCM formulas.

### Adjustment of TCM prescriptions to cancers of the lungs, GI tract and female reproductive systems in Taiwan in winter

**Figure 5:**
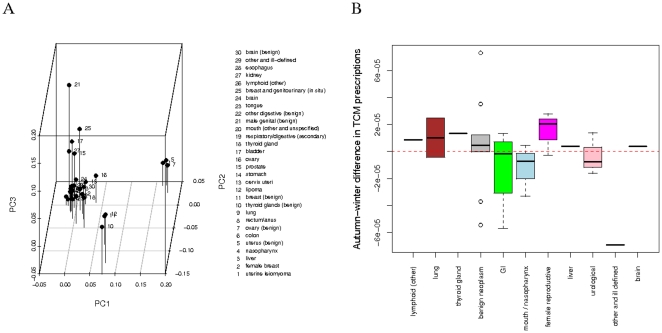
Variations in TCM prescriptions between seasons. (A) A cancer prescription is represented by a point in the three dimensional space spanned by the first three PCs, that together account for 55% of the data variation as shown in Figure S7. The points come from prescriptions made in spring. Figures S8, S9, and S10 show the prescriptions made in summer, autumn and winter, in the space defined by the same three PCs. (B) Differences between autumn- and winter-prescriptions are plotted against cancers. Cancer color codes follow those of Figure 2.

We divided the raw data into four parts: spring (March, April, May), summer (June, July, August), autumn (September, October, November) and winter (December, January, February) based on the dates when the diagnoses and prescriptions were made. Variability in treatment can be due to patient genotypes, TCM doctors, and/or seasons. To remove minor fluctuations (due, e.g., to different TCM clinics) while retaining most of the variances (due, e.g., to different cancer types and seasonal effects) in the data, we employed PCA on the 746 by 746 covariance matrix of the 746 by 120 herb-cancer-season weight matrix (see Material and Methods for details on PCA). In the analysis, formulas/herbs prescribed to a cancer diagnosed in spring are considered potentially different from those to the cancer diagnosed in summer, etc. The season-wise cancer-specific TCM prescriptions are shown as points in the first three PC space in Figures 5A and S8, S9, and S10. From the displacement of points between seasons, the between-season prescription changes were calculated in Figure S11, showing that autumn to winter displays the most changes in the prescriptions. On the other hand, the season-wise mean temperatures, relative humidities and precipitations of Taiwan, available from the government [28] and shown in Figures S12, S13, and S14, indicate that the largest temperature change in Taiwan happens between autumn ( = 25 degree Celsius) and winter ( = 18 degree Celsius) and that both relative humidities and precipitations dramatically change from summer to autumn. The autumn-winter prescription changes are stratified over the cancer types in Figure 5B. We conclude that TCM treatments for cancers of the lungs, gastrointestinal tract and female reproductive system are subject to adjustment in Taiwan in winter when the average temperatures drop.

## Discussion

Environmental changes induce physiological responses. Therapies that take into account interactions between the environment and the individual are conceived to be more productive. Among changes in the three meteorological variables, only drop in temperatures was found to correlate temporally with the changes in prescription. According to Figures S12, S13, and S14, Taiwan summer is both hottest and dampest while Taiwan winter is coldest but not necessarily driest. If prescriptions were adjusted to drop in temperatures in winter, it would be expected that they be adjusted to rise in temperatures and humidities in summer too. One explanation is that most Taiwan households are equipped with air-conditioners. Indoor conditions in summer are therefore not as hot and humid as the records show. On the other hand, Taiwan winter is cold both indoor and outdoor as typical Taiwan households are not equipped with heaters. Lungs could be affected as they take in cold airs. The digestive and female reproductive systems might need to adjust as the body needs more energy and blood in cold days. Our analysis of the TCM prescriptions to cancers in Taiwan therefore identifies the relevant environmental factor, organs and patients for better care.

TCM theories hold that imbalance or disharmony in the interactions among the functional elements in the body or in the interaction between the body and the environment lead to disease. TCM is allopathic like modern western medicine. It is therefore interesting to learn, from the prescription data, what cancers are in TCM perspective. *Peptic* and *tranquilizing* formulas are relatively few in the repertoire of reimbursable TCM formulas, ranking, respectively, 11^th^ and 16^th^ in the 21 TCM categories (see Table S1). However, in TCM prescriptions to cancer patients with stomach and sleep secondary disorders, the percentage weights of *peptic* and *tranquilizing* formulas were among the top three along with *tonifying* and *mediating* formulas. A sensible mapping between ICD-9 and TCM therapeutic categories seems to be established, helping dialogue between the two medicines. Furthermore, in the prescriptions to single, primary malignant cancers without comorbidities, *mediating* formulas rose to 2^nd^ heaviest from their 7^th^ position in the TCM categories of Table S1, in contrast to *tonifying* formulas, which, although the heaviest, are the most common (cf Table S1). *Tonifying* formulas, unlike *mediating*, are thus probably not peculiar in cancer treatment. According to TCM theories, *mediating* formulas are for the so-called *Shao Yang* syndromes which are neither *exterior* nor *interior*, and neither *cold* nor *warm*. The indeterministic nature of the TCM syndrome in regard to malignant cancers may be recapitulating the transformability and/or metastasizing of the disease.

Likewise, comparing TCM natures' weights in the cancer prescriptions with the natures' relative frequencies in the arsenal of the reimbursable herbs (*warm*, *cold*, *neutral*, *mild-cold*, *cool*, *mild-warm*, *hot*, in the order of their frequencies as shown in Table S2), we found predominance of *cold* or *mild-cold* TCM natures to neoplasms. The same comparison of Figure 3C and Table S3 leads to *pungent-bitter* TCM herbs in the TCM cancer prescriptions. Since, in TCM theories, *cold* herbs antagonize *warm* syndromes and *pungent* herbs move and disperse, the analysis may suggest that TCM views neoplasms as a *warm* and *stagnant* syndrome. As warmth and swelling are two of the features of inflammation, anti-inflammatory regimens such as exercise and toxin-free diets/environment may help prevent cancers. Indeed, the chemopreventive effect of non-steroidal anti-inflammatory drugs on colorectal and probably other cancers has been recognized and clinical trials for the evaluation of risks and benefits have been underway [29].

The ICD-9 codes in the study share a common denominator, that is, they are cancers with the same hallmarks at the cellular level. They differ otherwise, originating from different anatomical organs of different physiological functions. The design of the study helps address the issue of tissue-specificity of TCM prescriptions. Provided that the TCM treatments of cancers were efficacious, the result supports tissue-specificity of TCM prescriptions via weighted combinations of formulas and herbs. In western pharmaceutics, a targeted therapy drug which was approved for a cancer was later approved for other indications. The knowledge of TCM combinations may help inspire further indication expansion of targeted cancer drugs.

In linguistics, less specific words have higher frequencies. Use of low (high) specificity words, although appealing to speakers (hearers), incurs decoding (memory) cost of the hearers (speakers). A trade-off in the efforts between both parties was shown to lead to Zipf's distribution of words [30]. Abundances of expressed genes in human normal and cancer tissues were found to be Zipf-distributed [31]. In the chemical world, a recent study shows that the distributions of such features as rigid segments, ring systems and circular substructures of small, organic molecules follow power law [32]. The Zipf distribution of the weights of the TCM formulas and herbs may therefore suggest TCM treatment as a dialect, with herbs as words and formulas as phrases, in the communications to the human body. Note that Zipf distributions are considered a necessary but not sufficient condition for a language as ‘words’ in random texts were also found to exhibit Zipf distributions [33]. The Zipf-like distribution however may have implications in the dosage optimization of targeted cancer drug cocktails.

Modern western medicine is the major treatment modality in Taiwan. TCM patients might have received prior western cancer therapy or be under concomitant therapies. The information is not available in the dataset, nor is the information about prognosis of the TCM treatment. Interpretation can become diverse. For example, the most prescribed *tonifying* formulas and *sweet* herbs in TCM prescriptions could be aiming at fatigue, which is the most common side effect of radiation and chemotherapy. Despite the limitations, the systematic and exploratory analysis of the current study sheds light on TCM treatment of cancer, providing a fertile ground for the development of an integrated cancer management.

## Supporting Information

Figure S1 **Distribution of age.** Age distributions of those who have a single, cancer diagnoses without other secondary diagnoses.
(TIF)

Figure S2 **Distribution of cancer types.** The 3-digit numbers in blue are the ICD-9 codes of the cancers.
(TIF)

Figure S3 **Boxplot of Zipf exponents.** Exponents were the slopes from the linear regression to the first 20 datapoints, i.e. the 20 heaviest formulas/herbs, in the Zipf plots of individual cancers.
(TIF)

Figure S4 **Heatmap and hierarchical clustering of cancers and formulas/herbs.** Rows are formulas (in blue) or herbs (in green) and columns are cancers. Color key and histogram show the value and distribution of the weights.
(TIF)

Figure S5 **Distribution of age.** Age distributions for those who have, in addition to their primary cancer diagnoses, a secondary, but not tertiary, diagnosis.
(TIF)

Figure S6 **Distribution of secondary diagnoses.** The highest two account for 21% of all the secondary diagnoses.
(TIF)

Figure S7 **The first 10 eigenvalues of the first 10 eigenvectors (principal components) of the 746 by 746 covariance matrix from the 120 by 747 cancer-season-herb weight matrix.** The first 3 eigenvalues (i.e. variances) account for 55% of the sum of all the eigenvalues.
(TIF)

Figure S8 **Summer prescriptions to cancers.** A PC is a linear combination of formula/herb weights. The PCs are orthogonal to one another in the sense that they are uncorrelated. PCs are ordered such that data projected on the first PC show most variation, etc.
(TIF)

Figure S9 **Autumn prescriptions to cancers.** A PC is a linear combination of formula/herb weights. The PCs are orthogonal to one another in the sense that they are uncorrelated. PCs are ordered such that data projected on the first PC show most variation, etc.
(TIF)

Figure S10 **Winter prescriptions to cancers.** A PC is a linear combination of formula/herb weights. The PCs are orthogonal to one another in the sense that they are uncorrelated. PCs are ordered such that data projected on the first PC show most variation, etc.
(TIF)

Figure S11 **Difference in the TCM prescriptions between seasons.** Difference is calculated from the displacement of datapoints between two seasons by ∑_i_(PC_spring_−PC_summer_)_i_ * variance_i_, where PC_spring_ are the coordinates of the points in spring, variance_i_ is the variance (i.e. eigenvalue) of PCi and i = 1, 2, 3. Note that the spring in spring-winter is spring 2007, not spring 2008.
(TIF)

Figure S12 **Mean temperatures of Taiwan in different seasons.** Monthly thirty-year (1981–2010) mean temperatures in Celsius were taken for each of the four metropolises in Taiwan: Taipei, Taichung, Tainan and Kaohsiung. Pan-Taiwan monthly mean temperatures were calculated by averaging over the metropolises and then plotted by the seasons. Temperatures changed most from autumn to winter.
(TIF)

Figure S13 **Mean relative humidities of Taiwan in different seasons.** Monthly thirty-year (1981–2010) mean relative humidities were obtained for each of the four metropolises in Taiwan: Taipei, Taichung, Tainan and Kaohsiung. Pan-Taiwan monthly mean relative humidities were calculated by averaging over the metropolises and then plotted by the seasons. Relative humidities changed most from summer to autumn.
(TIF)

Figure S14 **Mean precipitations of Taiwan in different seasons.** Monthly thirty-year (1981–2010) mean precipitations were obtained for each of the four metropolises in Taiwan: Taipei, Taichung, Tainan and Kaohsiung. Pan-Taiwan monthly mean precipitations were calculated by averaging over the metropolises and then plotted by the seasons. Precipitations changed most from summer to autumn.
(TIF)

Table S1 **Categories of the TCM formulas.**
(DOC)

Table S2 **Natures of the TCM herbs.**
(DOC)

Table S3 **Flavors of the TCM herbs.**
(DOC)

## References

[1] WHO website.. http://www.who.int/mediacentre/factsheets/fs297/en/index.html.

[2] Hanahan D, Weinberg RA (2011). Hallmarks of Cancer: the Next Generation.. Cell.

[3] Gerber DE (2008). Targeted Therapies: a New Generation of Cancer Treatments.. Am Fam Physician.

[4] Aggarwal S (2010). Targeted cancer therapies.. Nat Rev Drug Discovery.

[5] Jemal A, Ward E, Thun M (2010). Declining Death Rates Reflect Progress against Cancer.. PLoS ONE.

[6] Miller VA, Kris MG, Shah N, Patel J, Azzoli C (2004). Bronchioloalveolar Pathologic Subtype and Smoking History Predict Sensitivity to Gefitinib in Advanced Non-Small-Cell Lung Cancer.. J Clin Oncol.

[7] Liu CC, Prior J, Piwnica-Worms D, Bu G (2010). LRP6 overexpression defines a class of breast cancer subtype and is a target for therapy.. Proc Natl Acad Sci U S A.

[8] Sawyers CL (2007). Cancer: mixing cocktails.. Nature.

[9] Unschuld PU (2003). Huang Di Nei Jing Su Wen: Nature, Knowledge, Imagery in an Ancient Chinese Medical Text.

[10] Zhang Z, Ye F, Wiseman N, Mitchell C, Feng Y (1999). Shang Han Lun: On Cold Damage, Translation and Commentaries.

[11] Wiseman N, Willms S, Ye F (2009). Jin Gui Yao Lue – Essential Prescriptions of the Golden Coffer.

[12] Harris ES, Cao S, Littlefield BA, Craycroft JA, Scholten R (2011). Heavy metal and pesticide content in commonly prescribed individual raw Chinese Herbal Medicines.. Sci Total Environ.

[13] Sung JJ, Leung WK, Ching JYL, Lao L, Zhang G (2004). Agreements among traditional Chinese medicine practitioners in the diagnosis and treatment of irritable bowel syndrome.. Aliment Pharmacol Ther.

[14] Zhang GG, Lee WL, Lao L, Bausell B, Berman B (2004). The variability of TCM pattern diagnosis and herbal prescription on rheumatoid arthritis patients.. Altern Ther Health Med.

[15] Patwardhan B, Warude D, Pushpangadan P, Bhatt N (2005). Ayurveda and traditional Chinese medicine: A comparative overview.. Evid Based Complement Alternat Med.

[16] NHRI website.. http://w3.nhri.org.tw/nhird/en/index.htm.

[17] Yang S-Z (1998). The Divine Farmer's Materia Medica: A Translation of the Shen Nong Ben Cao.

[18] Wu FM, Hsieh MH (2001). Collected Exegesis of Recipes, Wang Ang (1682).

[19] Hsieh HY, Chiu PH, Wang SC (2011). Epigenetics in traditional Chinese pharmacy: a bioinformatic study at pharmacopoeia scale.. Evid Based Complement Alternat Med.

[20] Wang S-C, Petronis A (2008). DNA Methylation Microarrays: Experimental Design and Statistical Analysis.

[21] R Development Core Team (2009). R: A language and environment for statistical computing. (Vienna, Austria): R Foundation for Statistical Computing..

[22] Yu MC, Yuan JM (2002). Epidemiology of nasopharyngeal carcinoma.. Seminars in Cancer Biol.

[23] Adamic L (2011). Complex systems: Unzipping Zipf's law.. Nature.

[24] Ha LQ, Sicilia-Garcia EI, Ming J, Smith FJ (2003). Extension of Zipf's law to words and phrases.. Proc 19th Intl Conf Comput Linguistics.

[25] Axtell RL (2001). Zipf Distribution of U.S. Firm Sizes.. Science.

[26] Hsieh SC, Lai JN, Lee CF, Hu FC, Tseng WL (2008). The prescribing of Chinese herbal products in Taiwan: a cross-sectional analysis of the national health insurance reimbursement database.. Pharmacoepidemiol Drug Saf.

[27] Li Shizhen (1578). Ben Cao Gang Mu.

[28] Central Weather Bureau website.. http://www.cwb.gov.tw/V7/climate/monthlyMean/Taiwan_tx.htm.

[29] Cuzick J, Otto F, Baron JA, Brown PH, Burn J (2009). Aspirin and non-steroidal anti-inflammatory drugs for cancer prevention: an international consensus statement.. Lancet Oncol.

[30] Ferrer-i-Cancho R, Sole RV (2003). Least effort and the origins of scaling in human language.. Proc Natl Acad Sci U S A.

[31] Furusawa C, Kaneko K (2003). Zipf's law in gene expression.. Phys Rev Lett.

[32] Benz RW, Swamidass J, Baldi P (2008). Discovery of Power-Laws in Chemical Space.. J Chem Inf Model.

[33] Li W (1992). Random Texts Exhibit Zipf's-Law-Like Word Frequency Distribution.. IEEE Transactions on Information Theory.

