# Supplementary figures and images for: Prescriptions of Traditional Chinese Medicine Are Specific to Cancer Types and Adjustable to Temperature Changes
Source: PLoS One. 2012 Feb 16;7(2):e31648. doi: 10.1371/journal.pone.0031648 (PMC3280982; doi:10.1371/journal.pone.0031648)

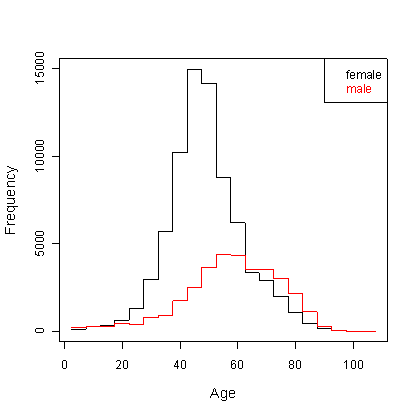

Supplement: Figure S1 — Distribution of age. Age distributions of those who have a single, cancer diagnoses without other secondary diagnoses. (TIF) [file pone.0031648.s001.tif]

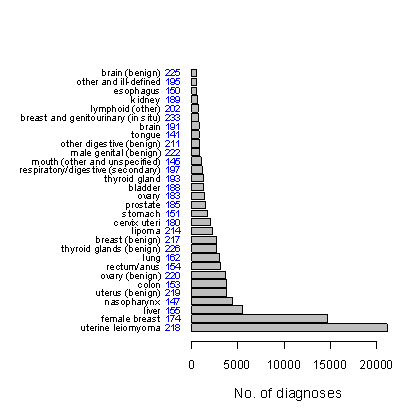

Supplement: Figure S2 — Distribution of cancer types. The 3-digit numbers in blue are the ICD-9 codes of the cancers. (TIF) [file pone.0031648.s002.tif]

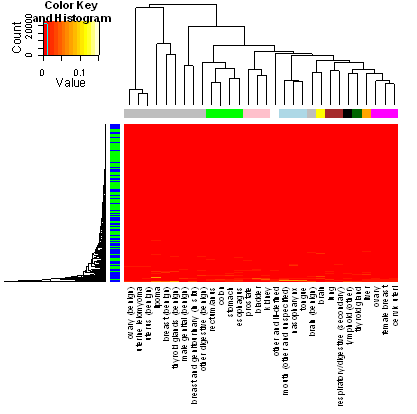

Supplement: Figure S4 — Heatmap and hierarchical clustering of cancers and formulas/herbs. Rows are formulas (in blue) or herbs (in green) and columns are cancers. Color key and histogram show the value and distribution of the weights. (TIF) [file pone.0031648.s004.tif]

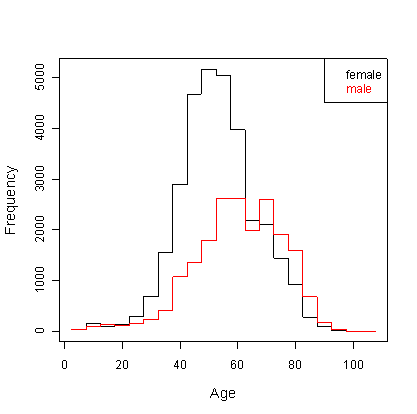

Supplement: Figure S5 — Distribution of age. Age distributions for those who have, in addition to their primary cancer diagnoses, a secondary, but not tertiary, diagnosis. (TIF) [file pone.0031648.s005.tif]

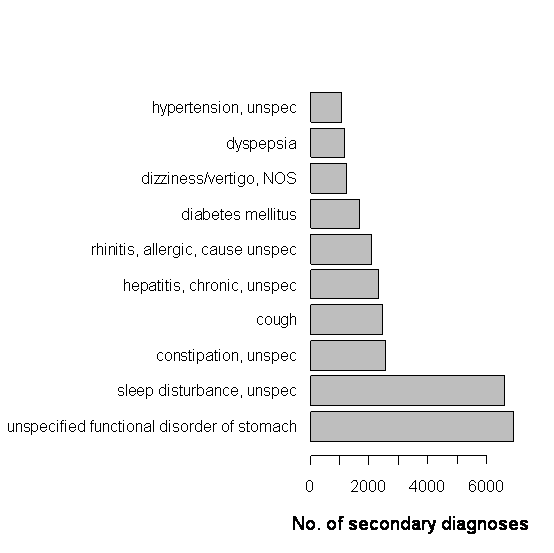

Supplement: Figure S6 — Distribution of secondary diagnoses. The highest two account for 21% of all the secondary diagnoses. (TIF) [file pone.0031648.s006.tif]

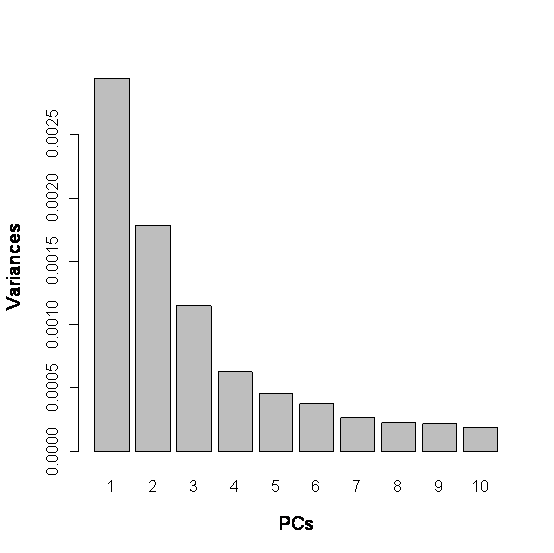

Supplement: Figure S7 — The first 10 eigenvalues of the first 10 eigenvectors (principal components) of the 746 by 746 covariance matrix from the 120 by 747 cancer-season-herb weight matrix. The first 3 eigenvalues (i.e. variances) account for 55% of the sum of all the eigenvalues. (TIF) [file pone.0031648.s007.tif]

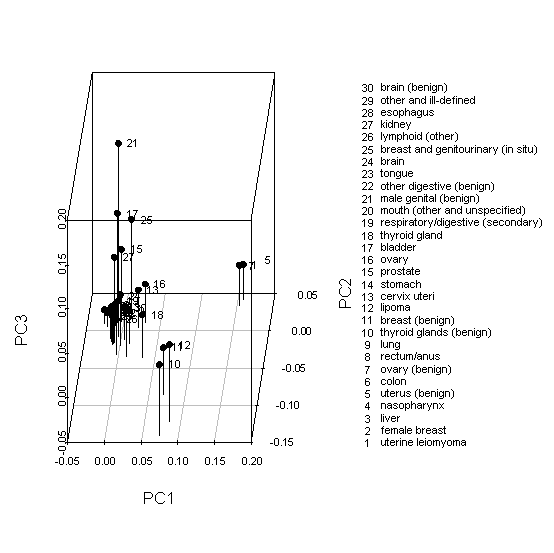

Supplement: Figure S8 — Summer prescriptions to cancers. A PC is a linear combination of formula/herb weights. The PCs are orthogonal to one another in the sense that they are uncorrelated. PCs are ordered such that data projected on the first PC show most variation, etc. (TIF) [file pone.0031648.s008.tif]

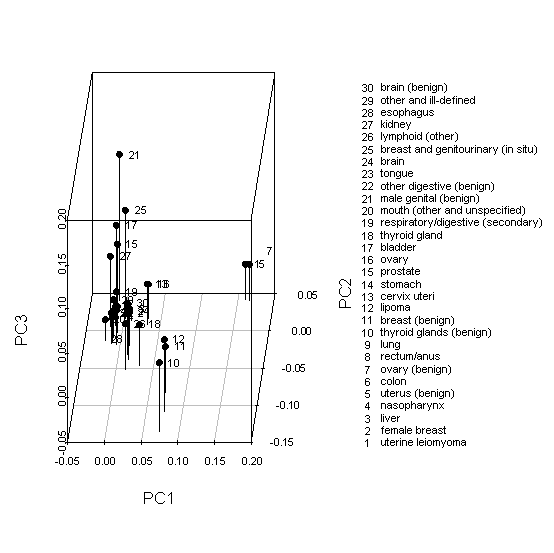

Supplement: Figure S9 — Autumn prescriptions to cancers. A PC is a linear combination of formula/herb weights. The PCs are orthogonal to one another in the sense that they are uncorrelated. PCs are ordered such that data projected on the first PC show most variation, etc. (TIF) [file pone.0031648.s009.tif]

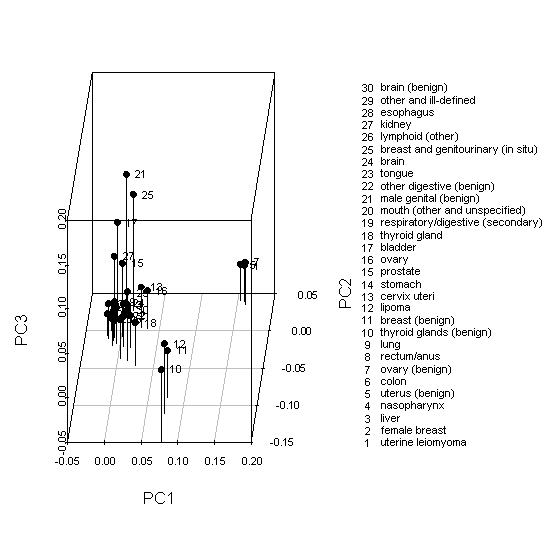

Supplement: Figure S10 — Winter prescriptions to cancers. A PC is a linear combination of formula/herb weights. The PCs are orthogonal to one another in the sense that they are uncorrelated. PCs are ordered such that data projected on the first PC show most variation, etc. (TIF) [file pone.0031648.s010.tif]

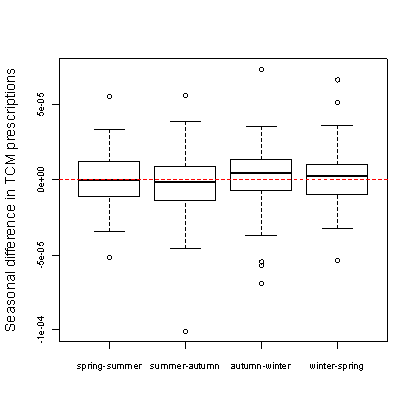

Supplement: Figure S11 — Difference in the TCM prescriptions between seasons. Difference is calculated from the displacement of datapoints between two seasons by ∑i(PCspring−PCsummer)i * variancei, where PCspring are the coordinates of the points in spring, variancei is the variance (i.e. eigenvalue) of PCi and i = 1, 2, 3. Note that the spring in spring-winter is spring 2007, not spring 2008. (TIF) [file pone.0031648.s011.tif]
