# Supplementary material for: Prescriptions of Traditional Chinese Medicine Are Specific to Cancer Types and Adjustable to Temperature Changes
Source: PLoS One. 2012 Feb 16;7(2):e31648. doi: 10.1371/journal.pone.0031648 (PMC3280982; doi:10.1371/journal.pone.0031648)
Supplement: Table S1 — Categories of the TCM formulas. (DOC) [file pone.0031648.s015.doc]

**Table S1: Categories of the TCM formulas***

| Rank | TCM formula effect | Number |
| --- | --- | --- |
| 1 | *tonifying* | 45 |
| 2 | *fire-purging* | 38 |
| 3 | *dampness-eliminating* | 24 |
| 4 | *blood-regulating* | 22 |
| 5 | *exterior-relieving* | 20 |
| 6 | *exterior-interior* | 19 |
| 7 | *mediating* | 19 |
| 8 | *phlegm-dissolving* | 18 |
| 9 | *wind-expelling* | 17 |
| 10 | *qi-regulating* | 14 |
| 11 | *peptic* | 14 |
| 12 | *carbuncle-treating* | 14 |
| 13 | *gynecologic* | 13 |
| 14 | *cold-dispelling* | 13 |
| 15 | *dryness-treating* | 12 |
| 16 | *tranquilizing* | 10 |
| 17 | *heat-clearing* | 9 |
| 18 | *astringing* | 9 |
| 19 | *interior-warming* | 6 |

*only 19 TCM categories are populated by the reimbursable formulas.
