# Supplementary material for: Prescriptions of Traditional Chinese Medicine Are Specific to Cancer Types and Adjustable to Temperature Changes
Source: PLoS One. 2012 Feb 16;7(2):e31648. doi: 10.1371/journal.pone.0031648 (PMC3280982; doi:10.1371/journal.pone.0031648)
Supplement: Table S2 — Natures of the TCM herbs. (DOC) [file pone.0031648.s016.doc]

**Table S2: Natures of the TCM herbs**

| Rank | TCM herb nature | Number |
| --- | --- | --- |
| 1 | *warm* | 107 |
| 2 | *cold* | 97 |
| 3 | *neutral* | 85 |
| 4 | *mild-cold* | 59 |
| 5 | *cool* | 28 |
| 6 | *mild-warm* | 25 |
| 7 | *hot* | 9 |
