# Supplementary material for: Prescriptions of Traditional Chinese Medicine Are Specific to Cancer Types and Adjustable to Temperature Changes
Source: PLoS One. 2012 Feb 16;7(2):e31648. doi: 10.1371/journal.pone.0031648 (PMC3280982; doi:10.1371/journal.pone.0031648)
Supplement: Table S3 — Flavors of the TCM herbs. (DOC) [file pone.0031648.s017.doc]

**Table S3:** Flavors of the TCM herbs

| Rank | TCM herb flavor | Number |
| --- | --- | --- |
| 1 | *sweet* | 54 |
| 2 | *bitter* | 49 |
| 3 | *pungent* | 47 |
| 4 | *pungent-bitter* | 33 |
| 5 | *bitter-pungent* | 31 |
| 6 | *sweet-bitter* | 22 |
| 7 | *pungent-sweet* | 18 |
| 8 | *sweet-bland* | 18 |
| 9 | *bitter-sweet* | 15 |
| 10 | *salty* | 11 |
| 11 | *sweet-bitter* | 9 |
| 12 | *bitter-astringent* | 9 |
| 13 | *bitter-sour* | 8 |
| 14 | *sweet-pungent* | 8 |
| 15 | *sweet-astringent* | 8 |
| 16 | *pungent-mild-bitter* | 7 |
| 17 | *sweet-salty* | 7 |
| 18 | *sweet-sour* | 6 |
| 19 | *sour-astringent* | 4 |
| 20 | *sour* | 4 |
| 21 | *salty-sweet* | 3 |
| 22 | *bitter-sweet-astringent* | 3 |
| 23 | *sour-sweet* | 2 |
| 24 | *bland-bitter* | 2 |
| 25 | *mild-bitter-mild-pungent* | 2 |
| 26 | *bitter-salty* | 2 |
| 27 | *bitter-mild-sweet* | 2 |
| 28 | *mild-bitter* | 2 |
| 29 | *bitter-mild-pungent* | 2 |
| 30 | *pungent-sweet-mild-bitter* | 2 |
